# Supplementary material for: Complexome profiling on the Chlamydomonas lpa2 mutant reveals insights into PSII biogenesis and new PSII associated proteins
Source: J Exp Bot. 2021 Aug 26;73(1):245–62. doi: 10.1093/jxb/erab390 (PMC8730698; doi:10.1093/jxb/erab390)
Supplement: erab390_suppl_Supplementary_Dataset_S1 [file erab390_suppl_supplementary_dataset_s1.zip › Supplemental Dataset 1 - Excel List and all profiles/plots/FKB16-5_Cre16.g675500.html]

Trivial name: FKB16-5  
  
Euclidean distance: 14332.19  
Mean Intensity (WT): 1227.50  
Mean Intensity (Mut): 1802.42  
Distance: 7.95  
  
MapMan: cell.cycle.peptidylprolyl isomerase;protein.assembly and cofactor ligation;protein.folding.immunophilins (IMM).FKBPs  
  
p value of intensity sums Welch test: 0.6354
